# Gastric Digestion and Changes in Serum Amino Acid Concentrations after Consumption of Casein from Cow and Goat Milk: A Randomized Crossover Trial in Healthy Males

**DOI:** 10.1016/j.tjnut.2025.07.025

**Published:** 2025-08-05

**Authors:** Elise JM van Eijnatten, Guido Camps, Wolf Rombouts, Linette Pellis, Paul AM Smeets

**Affiliations:** 1 Division of Human Nutrition and Health, Wageningen University, Wageningen, The Netherlands; 2 Ausnutria Dairy Corporation Ltd, Zwolle, The Netherlands

**Keywords:** milk protein, amino acids, coagulation, stomach, bovine, caprine, digestion, magnetic resonance imaging

## Abstract

**Background:**

In vitro studies show that goat milk proteins form less compact coagulates in the stomach compared with cow milk proteins, which may facilitate gastric digestion and amino acid (AA) absorption. However, this has not been confirmed in vivo in humans.

**Objectives:**

This study aims to examine gastric digestion and changes in serum AA concentrations after cow milk-derived (cow MC) and goat milk-derived casein (goat MC) ingestion.

**Methods:**

In this single-blind randomized crossover study participants consumed 300 mL of a drink containing 30 g of cow MC or goat MC. Participants underwent gastric magnetic resonance imaging (MRI) scans at baseline and every 10 min ≤60 min postprandially. Blood was drawn at baseline and ≤4 h postprandially. In addition, participants verbally rated their appetite after each MRI measurement. Primary outcomes were gastric emptying and serum AA concentrations. Secondary outcome was gastric coagulation as inferred by image texture metrics.

**Results:**

A total of 18 males (age 26 ± 8.3 y, body mass index 23 ± 1.6 kg/m^2^) completed the study. Gastric emptying half-time was 80 ± 25 min for goat and 85 ± 24 min for cow MC (*P* = 0.395). In line with this, gastric emptying of the drinks over time was similar [mean difference (MD) 0.77 mL; 95% confidence interval (CI): −6.9, 8.5; *P* = 0.845]. Serum essential AA (MD −110 μmol/L; 95% CI: −162, −58) was higher over time for cow MC (*P <* 0.001). The image texture metric contrast was lower for cow MC (MD 0.010; 95% CI: 0.001, 0.020; *P =* 0.036).

**Conclusions:**

Cow and goat MC have different coagulating properties, as inferred by AA concentrations and supported by image texture analysis. However, overall gastric emptying and the emptying of the liquid and coagulated fractions were similar. This warrants further in vivo research on casein coagulation in the food matrix to help determine the optimal use for cow and goat milk and their protein fractions.

This trial was registered at Netherlands Trial Registry as NL8137.

## Introduction

Protein is an essential macronutrient used in many processes in the human body [1,2]. It is important that ingested protein is properly digested and absorbed so that it can be used for protein synthesis [[3], [4], [5]]. Dairy products constitute a significant protein source globally [6]. Cow milk dairy products are the most commonly used, but goat milk popularity is increasing [7]. One of the reasons for this is the consumers’ perception of its health benefits. These benefits are hypothesized to originate from a difference in the milks’ digestion due to their different casein composition [8].

Cow and goat milk generally contain ∼3.5% protein of which caseins represent ∼80% and whey proteins ∼20% [9]. During digestion in the stomach the casein micelles (CM) are destabilized by pepsin proteolysis and acidification. This results in the formation of coagulates containing protein, and if present, fat globules [10]. The physical properties of these casein coagulates could affect gastric protein digestion, gastric emptying and subsequent intestinal digestion and absorption of amino acids (AA) [11]. Previous studies, predominantly in vitro, have shown that casein coagulation is affected by several factors including processing-induced protein modifications, overall product composition (food matrix), and differences in protein composition for instance between animal species [9,[12], [13], [14], [15], [16]]. Human in vivo studies on protein modifications, resulting in different cow casein forms, showed that coagulation and gastric emptying can have strong effects on the postprandial rise in AA bioavailability [17]. Even though goat micellar casein seemed to have similar protein structures on a molecular level in an in vitro study using X-ray scattering [18], CM differ in size, hydration, and mineralization compared with bovine caseins [8,19]. During static in vitro digestion, more β-casein and less αs1-casein may contribute to the formation of looser gastric clots in goat CM and therefore greater proteolysis than cow CM [20]. Increased access of the enzymes to the proteins might lead to faster gastric digestion of goat as compared with cow milk-derived caseins and milk, as seen in in vitro coagulum analysis [21]. In line with this, infant formula based on goat milk formed smaller flocs of aggregated protein and oil droplets under gastric conditions, leading to faster protein digestion in goat milk infant formula than seen in cow milk-based infant formula [22]. Similarly, goat milk proteins were digested faster than cow milk proteins in in vitro digestion by human gastric and duodenal enzymes [14]. This was also seen in 2 in vitro studies under simulated infant conditions where proteins in goat milk and goat milk-based infant formula had different digestive behavior compared with those present in cow milk and cow milk-based formula [23,24]. Maathuis et al. [23] controlled for gastric emptying. Therefore, differences in digestion were probably due to differences in coagulation. However, not all studies showed a faster digestion of goat milk protein. Inglingstad et al. [25] found in an in vitro study that more goat milk casein remained undigested after 30 min of digestion compared with cow milk casein. Because most research has been done in vitro and results are not conclusive, verification in vivo in humans is warranted. Understanding differences in coagulation and gastric emptying of cow and goat milk proteins is of interest because they may influence subsequent serum AA availability.

In summary, in vitro results suggest that the differences in digestion between cow and goat milk are due to differences in their casein. Therefore, the current in vivo study aimed to quantify the gastric digestion and absorption of cow and goat milk-derived casein in vivo in humans. We used magnetic resonance imaging (MRI) to evaluate intragastric processes and gastric emptying and examined blood AA concentrations. We hypothesized that goat milk-derived casein has different coagulum characteristics, faster gastric emptying, and higher serum AA concentrations compared with cow milk-derived casein.

## Methods

### Design

The study was a randomized crossover study in which healthy males underwent gastric MRI scans before and after consumption of 300 mL of a cow milk-derived-casein (cow MC) or goat milk-derived-casein (goat MC) drink. The primary outcome was gastric emptying [measured by gastric emptying half-time (GE-t50) and gastric volume over time]. Secondary outcomes were gastric coagulation, serum AA concentrations, serum glucose, insulin, free fatty acids (FFA), and triglyceride (TG) concentrations. In addition, appetite ratings (hunger, fullness, desire to eat, prospective consumption, and thirst) were measured. The study was approved by the ethical committee of Wageningen University with reference number NL70239.081.19, dated 19 September, 2019, conducted according to the principles of the Declaration of Helsinki (October 2013) and registered with the Dutch Trial Registry under number NL8137 (accessible through https://trialsearch.who.int/Trial2.aspx?TrialID=NL-OMON28580). We note that the trial registration mistakenly lists serum AA concentration as a coprimary outcome, but in the study protocol (NL70239.081.19) it was listed as a secondary outcome. All participants signed written informed consent.

### Sample size

In the study protocol, sample size was calculated based on the primary outcome, gastric emptying, as follows: given our previous work on gastric emptying of caloric liquids in adults [26], we know that the gastric emptying half-time of 500 mL of a dairy-based shake has an overall gastric emptying half-time (GE t50) of 54.7 ± 3.8 min. A thin 100-kcal shake had the lowest GE t50 of 26.5 ± 3.0 min, followed by a thick 100-kcal shake with a GE t50 of 41 ± 3.9 min, a thin 500-kcal shake with a GE t50 of 69.5 ± 5.9 min, and a thick 500-kcal shake with a GE t50 of 81.9 ± 8.3 min. The low and high energy shakes contained 0.2 kcal/mL and 1 kcal/mL, respectively.

The goat MCC contains 0.67 kcal/mL. Therefore, we expect an SD of 4.5 min for both drinks based on nutrient density. In Camps et al. [26], the average difference between 500-mL thin and thick shake’s GE t50 was 14.5 (low-cal) and 12.4 min (high-cal). For a 350-mL load, the GE will be smaller. As a crude assumption, we use a linear decline and take 0.7 times the difference in GE t50’s (10.15 and 8.68 min) and note that the drinks are fairly high-caloric. Thus, we estimated an average difference between the treatments of 9 min if the viscosity difference would be similar to that in Camps et al. [26]. However, because the effective viscosity difference is likely smaller in the current study (initial viscosity is the same), we took 50% of that, that is, 4.5 min as the difference in GE t50 between the treatments that we wanted to be able to detect. Combined with the SD of 4.5, a significance level of 0.05 (2-sided) and a power of 0.8, 18 research subjects are required.

Due to small changes in the treatment formulation the goat MC drinks energy density became slightly lower (0.58 kcal/mL) so we verified the sample size with adjusted estimates as follows, arriving also at *n* = 18: we know that the GE-t50 of 500 mL dairy-based shakes differing in energy density and viscosity (protein content either 6 or 30 g and energy density 0.2 kcal/mL or 1 kcal/mL) has an overall GE-t50 of 54.7 ± 3.8 min, with the different shakes ranging between 26.5 ± 3.0 min and 81.9 ± 8.3 min. The goat MC drink contains 0.58 kcal/mL. On the basis of nutrient density, the expected SD of the cow MC and the goat MC drinks should be somewhere between 3 and 6. Therefore, we assumed an SD of 6 min for both drinks. In Camps et al. [26], the average difference in GE-t50 between a 500-mL thin and thick shake was 14.5 (low-calorie) and 12.4 min (high-calorie) [26]. For a 300-mL load, the GE-t50 will be smaller. We considered a 4-min difference in GE t50 between the treatments as the minimum detectable difference. It was noted later as part of the review process that for this updated sample size estimation which also yielded *n* = 18 participants inadvertently a calculator for a parallel study design was used. When using the appropriate crossover sample size calculator (http://hedwig.mgh.harvard.edu/sample_size/js/js_crossover_quant.html) that was also used in the study protocol with the following parameters for GE-t50: SD of the difference = 6 min, number of patients = undefined, power = 0.8, difference in means = 4 min the calculated total number of patients was 20. Thus, the target sample size estimate for the shakes used in the study should have been *n* = 20 instead of *n* = 18.

### Participants

Healthy males were recruited from November 2019 until March 2020 via e-mail using a database of individuals who expressed interest in participating in scientific research. Healthy, nonsmoking males with a BMI (in kg/m^2^) of 18.5–25 were included. Exclusion criteria were cow or goat milk allergy or lactose intolerance (self-reported), gastric disorders or regular gastric complaints, use of proton pump inhibitors or other gastric medication, or a contraindication to MRI scanning (including, but not limited to pacemakers and defibrillators, intraorbital or intraocular metallic fragments ferromagnetic implants or being claustrophobic).

### Treatments

Treatments were 300 mL drinks containing 30 g of protein originating from cow micellar casein concentrate powder (FrieslandCampina Refit MCI80) or 30 g goat micellar casein concentrate powder (Ausnutria Dairy Corporation Ltd., pilot plant). The drinks were matched on caloric content, protein, lactose, and dry matter content. The amount of the AA leucine was equal. Vanilla extract was added to ensure similarity of taste. The drinks were prepared by slowly reconstituting the caseins at 50°C and thereafter allowing them to fully rehydrate at 4°C overnight using a magnetic stirrer. This method assured a more similar viscosity in the drinks (when resolving fast by shaking, there was a larger viscosity of the cow MC drink: 200–500 mPa·s, and goat MC drink 8–15 mPa·s). Three samples of each drink were measured with a rheometer to assess their viscosity using an Anton Paar Physica MCR 301 rheometer equipped with a Couette geometry with a volume of 1 mL. Viscosity was determined at constant shear rate of 100 s^-1^ at 20°C. The difference in viscosity of the drinks (15.9 ± 3.9 mPa·s for the cow MC drink and 7.2 ± 1.8 mPa·s for the goat MC drink) was deemed not physiologically relevant (for instance milk’s viscosity is 2 mPa·s whereas that of yoghurt is 150 mPa·s which is a much larger difference).

The composition per 300 mL serving of the cow MC drink was 176 kcal, 30 g protein, 90:10 casein-to-whey ratio, 12 g lactose, 0.77 g fat, and 81 g dry matter. For the goat MC drink, this was 174 kcal, 30 g protein, 90:10 casein-to-whey ratio, 12 g lactose, 0.60 g fat, 81 g dry matter. The AA profiles of the drinks can be found in Table 1, and their components can be found in Supplemental Table 1. The data were analyzed by Qlip B.V. (lactose, dry matter, fat, protein) and NIZO (casein-to-whey ratio of the goat micellar casein concentrate; for cow micellar casein concentrate the 90:10 casein-to-whey ratio was stated on the product data sheet).

**TABLE 1:** Amino acid profile of cow and goat milk-derived casein drinks (g AA per 300 mL).^1^

AA type	Amino acid	Cow milk-derived casein drink	Goat milk-derived casein drink
Essential	Isoleucine	1.58	1.49
	Leucine	2.96	2.96
	Valine	1.61	1.60
	Histidine	0.93	0.85
	Lysine	2.60	2.61
	Methionine	0.87	0.80
	Phenylalanine	1.61	1.60
	Threonine	1.33	1.45
	Tryptophan	0.41	0.43
Total		13.9	13.8
Nonessential (or conditionally essential)	Arginine	1.14	0.89
	Cysteine	0.19	0.25
	Glutamine	6.86	6.24
	Glycine	0.58	0.50
	Proline	3.47	3.76
	Tyrosine	1.68	1.30
	Alanine	0.98	0.94
	Aspargine	2.24	2.19
	Hydroxyproline	<0.05	<0.05
	Ornithine	<0.05	<0.05
	Serine	1.78	1.50
Total		18.9	17.6

Abbreviation: AA, amino acid.

1 Analysis was performed by Eurofins Analytico B.V.

### Study procedures

**FIGURE 1:**
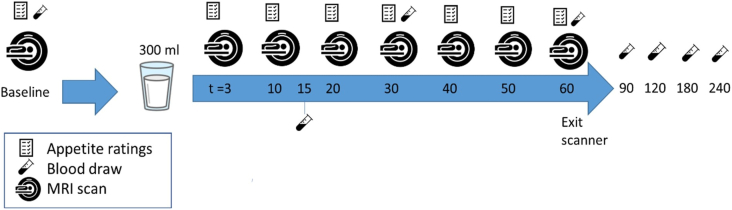
Overview of a test session.

Participants arrived after an overnight fast. Eating was allowed until 20:00 on the day before the study. Drinking water was allowed ≤1 h before the visit. Participants started their scan session at 8 or at 10:00 and were measured at the same time on both study days. First, a canula was placed in an antecubital vein. Then, participants verbally provided baseline appetite ratings after which the baseline MRI scan was performed and the baseline blood sample was drawn. Subsequently, they consumed 1 of the 300-mL drinks within 2 min. Participants were randomly allocated by block randomization using 9 blocks of 2 participants with AB or BA sequence using https://randomizer.org to receive either the cow or goat MC first. Participants were blind to which drink they received. Gastric MRI scans were performed at baseline and at *t* = 3, 10, 20, 30, 40, 50, and 60 min after the start of ingestion. During the MRI session, participants verbally rated hunger, fullness, prospective consumption, thirst, and nausea on a scale from 0 (not at all) to 100 (most imaginable) at each time point [27,28]. An overview of a test session is given in Figure 1.

### MRI

Participants were scanned in a supine position with the use of a 3 Tesla Siemens Verio MRI scanner (Siemens AG) using a T_2_-weighted spin echo sequence (HASTE, 24 6-mm slices, 2.4 mm gap, 1.19 × 1.19 mm in-plane resolution, TR 850 ms, TE 87 ms, flip angle 112°C) with breath hold command on expiration to fixate the position of the diaphragm and the stomach. The duration of the scan was ∼18 s. The software Medical Imaging Processing And Visualization (MIPAV, version 11.0.3) [29] was used to manually delineate gastric content on each slice and create a corresponding mask image. Gastric content volume on each time point was calculated by multiplying the surface area of gastric content per slice with slice thickness including gap distance, and summing up the volumes of all slices showing gastric content. In Matlab (version R2023a, multitresh function), a bias field correction was performed using multiplicative intrinsic component optimization [30]. To assess changes in gastric coagulation, image texture analysis of the stomach content was performed using the masks in the software LIFEx (version 7.2.0, Institut national de la santé et de la recherche médicale) [31]. Homogeneity, coarseness, contrast, and busyness were calculated. These image metrics provide information on the spatial patterns of voxel intensity [32]. The Gray-Level Co-occurrence Matrix method was used for homogeneity (degree of similarity between voxels), and Neighborhood Gray-level Difference Matrix difference of gray levels between 1 voxel and its 26 neighbors in 8 dimensions was used for contrast (local variations), coarseness (spatial rate of change in intensity), and busyness (spatial frequency of changes in intensity). The number of gray levels for texture metric calculation was set at 64, intensity rescaling at relative (ROI: min/max), and dimension processing at 2D. On each postprandial time point, texture metrics were calculated per slice for the stomach content. Subsequently, a weighted average texture metric was calculated based on the gastric content volume in each slice such that slices with little stomach content contributed less to the average than those with more stomach content. To quantify the (relative) volume of liquid and semisolid stomach contents the number of lighter (more liquid), intermediate and darker (semisolid) voxels was calculated by determining 2 intensity thresholds with the use of Otsu’s method [33] in Matlab, an approach previously used on in vitro and in vivo MRI images of gastric milk digestion [[34], [35], [36]]. The number of intermediate and darker voxels were summed and interpreted as reflecting voxels in which coagulation took place. This was done because visual inspection of the thresholding results using 1 threshold showed a poorer separation between lighter and darker gastric contents. In the context of this study, changes in image texture metrics were interpreted as reflecting changes in the degree of coagulation. An example of stomach contents with and without coagulation and the corresponding image texture metrics can be found in Supplemental Figure 1.

### Blood parameters

Blood samples were collected at baseline and at *t* = 15, 30, 60, 90, 120, 180, and 240 min in sodium-fluoride (for glucose), serum (for AA, FFA, and insulin), and lithium-heparin (for triglyceride) tubes. After collection, the blood in the serum tube was allowed to clot for 30 min. Subsequently, all tubes were centrifuged at 1300 ×*g* for 10 min at 20°C. After centrifugation, the supernatant was divided into aliquots and stored in a −80°C freezer until analysis. AA, FFA, and insulin concentrations were measured at Wageningen University. Glucose and TG concentrations were measured at the clinical chemistry laboratory of the Gelderse Vallei hospital.

AA concentrations were determined using triple quadrupole mass spectrometry, with an internal standard and ^13^C reference mix. Glucose concentrations were determined using an Atellica CH Glucose Hexokinase_3 (GluH_3) assay kit and Atellica CH analyzer (Siemens Healthineers). The lower limit of detection (LLOD) was 0.2 mmol/L, and intra-assay coefficients of variations (CVs) were at most 4.5%. Serum insulin concentrations were determined using an enzymatic immunoassay kit (ELISA, Mercodia AB) with an LLOD of 0.008 mmol/L and intra-assay CVs of a maximal 6.9%. Serum FFA concentrations were determined using an enzymatic assay kit (Instruchemie) with an LLOD of 4 mg/dL and intra-assay CVs of at most 1.4%. TG concentrations were quantified using an Atellica CH TG enzymatic assay kit and quantified using an Atellica CH analyzer (Siemens Healthineers) with a LLOD of 8 mg/dL and intra-assay CVs of at most 1.0%.

### Statistical analysis

To estimate GE-t50, a commonly used summary measure, a curve was fitted to the gastric volume over time of the cow MC and goat MC drink using R statistical software according to the linear-exponential model as developed on the basis of earlier models [37,38]. Further analyses were performed in SPSS (version 22, IBM). Essential amino acid (EAA), nonessential amino acid (NEAA), and branched-chain amino acid (BCAA) concentrations were calculated by adding individual AA concentrations. GE-t50 was compared between cow MC and goat MC drinks with a paired t-test. Normality was confirmed by inspecting QQ plots of the residuals. Overall gastric volume, coagulation (image texture of the gastric contents as reflected in homogeneity, contrast, coarseness, and busyness), blood parameters, and appetite ratings over time were evaluated using linear mixed models with “treatment,” “time,” and “treatment”∗“time” as fixed factors and baseline values as a fixed effect covariate. “Participants” was included as a random factor. The correlation structure assumed for the random factor “participants” was variance components. Post-hoc t-tests were performed with Tukey’s honestly significant difference (HSD) correction when there were significant effects, which were EAA, NEAA, BCAA, and the texture metric “contrast.” In addition, Pearson correlation coefficients were calculated for the associations between image texture metrics at 30 min (chosen because that was the first time point that coagulation was clearly visible) and GE-t50 and blood parameters (4-h AUC of EAA, NEAA, BCAA, glucose, and insulin). The significance threshold was set at *P* = 0.05. Data are expressed as mean ± SEM unless stated otherwise.

## Results

Eighteen healthy men (age 26 ± 8.3 y, BMI 23 ± 1.6) participated in the study. The flow diagram can be found in Supplemental Figure 2. Baseline characteristics of participants are shown in Table 2. An overview of all individual timepoints of each result can be found in Supplemental Table 2.

**TABLE 2:** Mean ± SD baseline characteristics of the participants for each treatment day (*n* = 18)

Characteristic	Value	Cow MC drink	Goat MC drink	Mean difference (95% CI)
Age (y)	26 ± 8.3			
BMI (kg/m^2^)	23 ± 1.6			
Gastric juice (mL)		19 ± 11	21 ± 10	1.9 (−0.8, 4.6)
Essential amino acids (μmol/L)		1607 ± 302	1576 ± 321	−31 (−109, 48)
Nonessential amino acids (μmol/L)		2102 ± 561	2044 ± 617	−58 (−207, 90)
Branched-chain amino acids (μmol/L)		979 ± 188	971± 190	−7 (−55, 40)
Glucose (mmol/L)		5.4 ± 0.4	5.4 ± 0.5	0.06 (−0.05, 0.2)
Insulin (mIU/L)		7.3 ± 5.5	7.2 ± 3.5	−0.09 (−1.2, 1.1)
Triglycerides (mmol/L)		0.86 ± 0.25	0.94 ± 0.37	0.08 (−0.01, 0.17)
Free fatty acids (mmol/L)		0.39 ± 0.24	0.33 ± 0.21	−0.06 (− 0.11, −0.002)
Hunger (points)		51 ± 20	51 ± 23	−0.3 (−5.8, 5.1)
Thirst (points)		56 ± 24	55 ± 24	−1.2 (−7, 5)
Fullness (points)		27 ± 21	29 ± 23	1.7 (−3.8, 7.1)
Desire to eat (points)		70 ± 15	63 ± 18	−7.5 (−12, −3)
Prospective consumption (points)		69 ± 18	65 ± 19	−3.3 (−8.0, 1.5)

Abbreviation: MC, micellar casein.

### Gastric emptying

**FIGURE 2:**
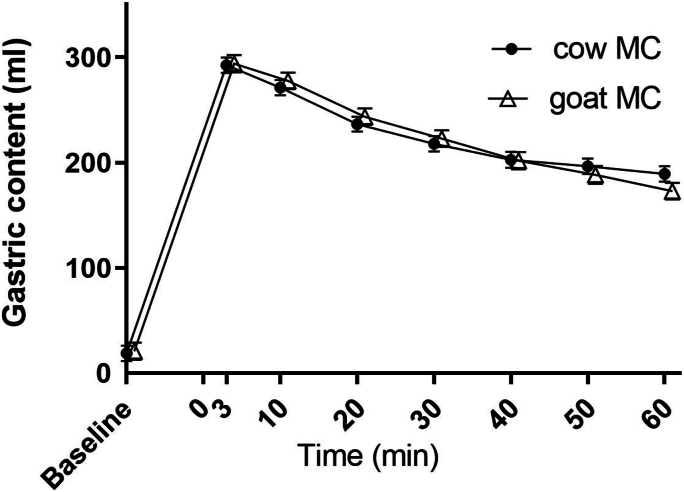
Mean ± SEM gastric content volume over time for the 300 mL cow (cow MC) and goat (goat MC) and milk-derived casein drinks. *T* = 0 min is the start of ingestion. A linear mixed model analysis showed no significant difference between the drinks. MC, micellar casein.

The curves of gastric emptying over time of the cow MC and goat MC drink are shown in Figure 2. GE t50 was 84.6 ± 23.7 min for the cow MC drink, compared with 79.8 ± 24.7 min for the goat MC drink (*P* = 0.395). The mixed model analysis showed no difference in gastric emptying over time between the drinks [mean difference (MD) 0.77; 95% confidence interval (CI): −6.9, 8.5; *P* = 0.845] and there was no time∗treatment interaction effect (*P* = 0.65). There was a significant effect of time (*P* < 0.001).

An example of a thresholded stomach image can be found in Supplemental Figure 3. The percentage of liquid and coagulum volume did not differ between the cow MC and goat MC drink over time (coagulum: MD 0.407%; 95% CI: −1.2, 2.0; *P* = 0.607), and there was no treatment∗time interaction effect (*P* = 0.52). There was a significant decrease in coagulum volume over time (time effect *P* = 0.002).

### Amino acids

**FIGURE 3:**
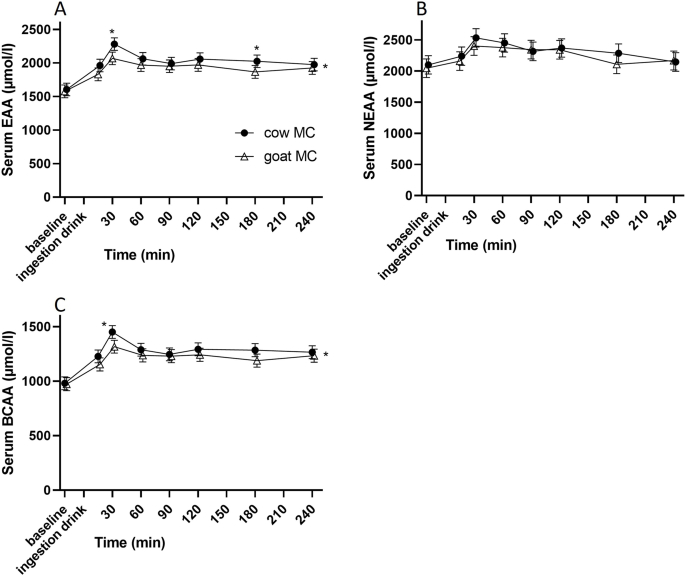
Mean concentration ± SD of (A) serum essential amino acid, (B) nonessential amino acid (NEAA), and (C) branched-chain amino acid (BCAA) concentrations after cow (cow MC) and goat (goat MC) milk-derived casein drink ingestion. ∗*P* < 0.05 placed at the right of the graph denotes a significant treatment effect. Above a data point, it denotes a significant time point (post-hoc t-test). EAA, essential amino acids; MC, micellar casein.

Figures of serum EAA, NEAA, and BCAA can be found in Figure 3. Figures of all individual AAs can be found in Supplemental Figures 4 and 5. Serum concentration of EAA over time was lower for goat MC (MD–110 μmol/L; 95% CI: −162, −58; *P* < 0.001). Post-hoc *t*-test showed that this was driven by time points *t* = 30 (*P* = 0.002) and *t* = 180 (*P* = 0.025) and that there tended to be a lower serum concentration of NEAA for goat MC over time (MD −62.1 μmol/L; 95% CI: −127, 3.4; *P* = 0.063), driven by time point *t* = 180 min (*P* = 0.045). Serum concentration of BCAA was also lower for goat MC (MD −65 μmol/L; 95% CI: −101, −29; *P* < 0.001). This was driven by time point *t* = 30 (*P* = 0.005), and there was a trend at *t* = 180 (*P* = 0.051). Time effects where significant for EAA, NEAA, and BCAA (*P* < 0.001). There were no interaction effects.

### Coagulation

**FIGURE 4:**
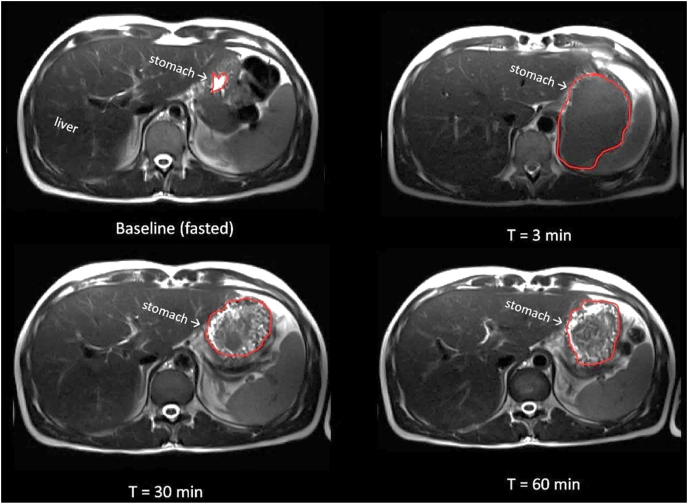
Examples of T_2_-weighted MRI images showing cross-sections through an empty stomach after an overnight fast (baseline) and after 300 mL casein drink consumption. At *t* = 30 and 60 min, coagulation can be observed. MRI, magnetic resonance imaging.

Figure 4 shows examples of MRI images at the level of the stomach showing homogenous stomach content after casein drink consumption and subsequent coagulum formation.

**FIGURE 5:**
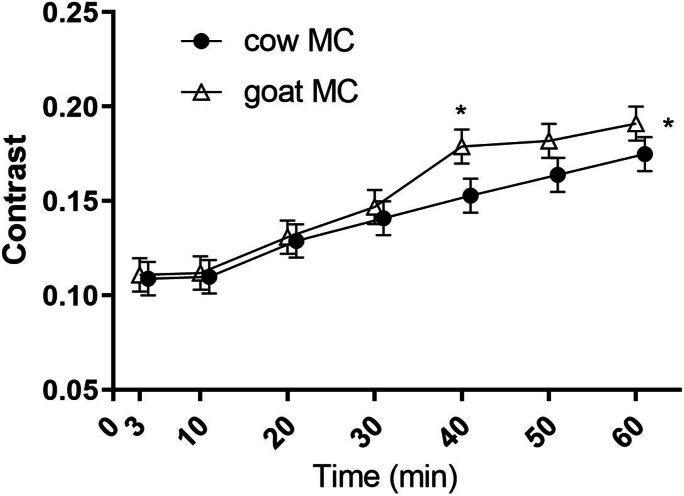
Mean ± SEM of the image texture metric contrast calculated over the stomach content over time after cow (cow MC) and goat (goat MC) milk-derived casein drink ingestion. A linear mixed model analysis showed a significantly higher contrast of goat MC (*P* = 0.036) driven by *t* = 40 min as seen with a post-hoc *t*-test. A higher contrast reflects a greater degree of structure in the image, which we interpret as a difference in coagulation. MC, micellar casein.

The curves of the image texture metric contrast of the stomach content over time can be found in Figure 5. The other image texture metrics can be found in Supplemental Figure 6. Contrast was significantly lower for the cow MC than for the goat MC drink (MD 0.010; 95% CI: 0.001, 0.020; *P* = 0.036). Homogeneity (MD −0.003; 95% CI: −0.012, 0.006; *P* = 0.503), coarseness (MD 0.001; 95% CI: 0.000, 0.001; *P* = 0.310) and busyness (MD −0.008; 95% CI: −0.023, 0.007; *P* = 0.315) were not significantly different between the drinks. Time effects where significant for all texture metrics (all *P* < 0.001). There were no interaction effects.

### Glucose and insulin

Overall, glucose concentrations did not differ between the drinks (MD −0.055 mmol/L; 95% CI: −0.14, 0.028; *P* = 0.19). Only when examining specific timepoints, glucose concentration for the goat MC drink was slightly higher compared with the cow MC drink at *t* = 30 min (MD 0.015 mmol/L; 95% CI: 0.42, 0.22; *P =* 0.036). Insulin concentrations did not differ between the cow MC and goat MC drinks (MD −1.66 mIU/L; 95% CI: 1.35, −0.16; *P* = 0.84) (Supplemental Figure 7). Time effects were significant for glucose and insulin (*P* < 0.001). There were no interaction effects.

### Triglycerides and free fatty acids

FFA concentrations were not different between the cow MC and goat MC drinks (MD −0.032 mmol/L; 95% CI: 0.034, 0.001; *P* = 0.948). TG concentrations over time were significantly higher for goat MC (MD 0.074 mmol/L; 95% CI: 0.039, 0.109; *P* = 0.009), even with baseline differences as a covariate in the mixed model analysis. However, there were no differences for any of the time points (see Supplemental Figure 8). Time effects were significant for FFA and TG (*P* < 0.001), and there were no interaction effects.

### Appetite ratings

Hunger and thirst did not differ between the treatments (*P* = 0.61 and 0.29, respectively). Fullness, desire to eat, and prospective consumption were significantly lower after goat MC ingestion (*P* = 0.036, *P* < 0.001, *P* < 0.001, respectively). However, as can be seen in Supplemental Figure 9, the differences are small (<10 units as a mean difference over time).

### Correlations between image texture metrics, blood concentrations, and gastric emptying

To explore the associations between image texture metrics as an indicator of the degree of coagulation, blood concentrations and gastric emptying, Pearson correlation coefficients were calculated for image texture metrics at 30 min (because at *t* = 30 min coagulation was visible) and 4-h AUC of blood parameters (EAA, NEAA, BCAA, glucose, and insulin). Overall, GE-t50 correlated negatively with contrast (*r* = −0.413; 95% CI: −0.662, −0.081) and coarseness (*r* = −0.38; 95% CI: −0.640, −0.043). NEAA correlated with insulin (*r* = 0.491; 95% CI: 0.152, 0.727). When divided between treatments: goat MC, EAA, NEAA and BCAA were negatively correlated with coarseness (*r* = −0.698; 95% CI: −0.887, −0.175; *r* = − 0.572; 95% CI: −0.837, −0.002; and *r* = −0.690; 95% CI: −0.891, −0.162, respectively). GE-t50 was positively correlated with insulin (*r* = 0.558; 95% CI: 0.130, 0.790). For cow MC GE-t50 was positively correlated with image homogeneity and busyness (*r* = 0.498; 95% CI: 0.061, 0.760; *r* = 0.568; 95% CI: 0.141, 0.787, respectively) and negatively correlated with contrast and coarseness (*r* = − 0.627; 95% CI: −0.822, − 0.217 and *r* = − 0.554; 95% CI: −0.780, − 0.134). All correlations can be found in Supplemental Figure 10.

## Discussion

This is the first study to explore the digestion of cow and goat MC by quantifying gastric emptying, gastric casein coagulation, and blood concentrations in humans. Overall, serum concentrations of EAA and BCAA were higher for cow MC. Gastric emptying curves and GE-t50 were similar for the cow MC and goat MC drinks. The image texture metric contrast was significantly different, suggesting a difference in coagulation. No significant differences between cow MC and goat MC were seen for serum insulin, glucose, and FFA concentrations. Serum TG concentrations were significantly higher in goat MC.

Coagulation was visible on the MRI scans for both drinks (Figure 4). In an attempt to objectively quantify this, image texture metrics were calculated for the stomach contents. A lower homogeneity and higher contrast could reflect “more” coagulation. Even though the actual meaning of differences in image texture metrics in reference to coagulating properties of stomach content requires more research, the difference in the metric ‘contrast’ does support the notion of a difference in coagulation between cow MC and goat MC. However, the metrics homogeneity, coarseness, and busyness did not differ between the drinks. It should be taken into account that higher homogeneity could not only reflect a more homogenous liquid, but also the presence of a large and fairly homogenous coagulum. Another aspect to consider is that not only the size, but also the structure is an important characteristic of coagulates. For instance, some coagulates can be firm and have a greater weight and denser structure than less dense coagulates with approximately the same volume [13]. Image texture metrics could possibly be used to quantify the density of the coagulum, as MRI can reflect the water content of the coagulum. Notably, MRI image texture parameters are affected by the resolution of the input images and could detect differences in image intensity patterns not appreciable by eye. This requires further validation by concomitant analysis of MRI images and coagulates that differ in size and density. To provide molecular-level information, other MRI techniques are being developed, such as measurement of the magnetization transfer ratio and relaxation rates [34,35,39,40]. These measurements require additional MRI measurements to be recorded, but could be used in follow-up research to examine more subtle differences in protein coagulation in vivo.

Cow MC and goat MC coagulates likely differ, because in vitro and animal in vivo research showed that goat milk coagulum had a softer consistency and less fused protein networks, especially toward a later stage of digestion [8,10]. We, therefore, hypothesized that this would lead to a longer gastric retention of coagulum and relatively faster GE for goat milk casein in comparison to cow milk casein. This is seen in recent human in vivo work [41]. However, we observed that the overall GE was similar, and the emptying of coagulum and liquid volume fraction did not differ between cow MC and goat MC. This is in line with recent findings that bovine milk coagulation differences did not affect GE, and GE did not explain differences in AA concentrations [42].

As expected, even though AA composition of the treatments were largely similar, postprandial serum AA concentrations differed between casein derived from goat and cow milk, which appears to be due in part to differences in their coagulation. Goat milks’ softer and smaller coagulates [21] would make the proteins more accessible to digestive enzymes (proteases) and lead to more efficient break down of peptide bonds such that goat milk proteins would be faster digested than cow milk proteins [14]. However, our study showed a higher total response of serum AA in the 5 h after cow milk consumption, which seems contradictive. On the other hand, in an in vitro study of Inglingstad et al. [25], who used a 2-step digestion model (gastric and duodenal), caseins from goat milk were less digested compared with caseins from cow milk during gastric digestion. Interestingly, in our study serum EAA concentrations were higher for cow MC, although we expected higher concentrations for goat MC based on in vitro studies [14,20]. However, we examined caseins in relative isolation where milk fat content was negligible, and the mineral profile was different than in the CM present in whole milk. The formation of coagulates interacts with minerals and fat and thus coagulation might be different for a whole food (that is in the “food matrix” [43]). For instance, the interaction of peptides with small fat globules can slow protein breakdown and thereby influence AA availability [44,45]. A second factor leading to differences in coagulation might be the difference in buffering capacity between cow and goat milk. Goat milk contains a higher nonprotein nitrogen (NPN) amount than cow milk and more NPN contributes to a slower acidification in the stomach [46]. Roy et al. [8] also discuss the high degree of variation in CM characteristics within the same species. Within and across species differences in breeds, genetic variants, and phosphorylation sites of the caseins may also add to the variation [47]. In summary, to our knowledge 3 factors could have contributed to the discrepancies of our study with other studies: *1*) the fact that this is the first human in vivo study on gastric digestion, *2*) that we assessed casein outside of the food matrix, *3*) the buffering capacity and the variation in casein characteristics even within species.

Fullness, desire to eat, and prospective consumption were significantly lower for goat MC, but these differences were small. For instance, the mean difference of desire to eat had a mean difference of 7 pts over the 60 measured minutes. A difference <10% is usually not considered as practically relevant [48]. Thus, this finding is in line with the lack of differences in gastric volume over time and coagulum volume over time between the drinks.

The strengths of this study were the single-blind, randomized, crossover study set-up in which measurements in different stages of digestion were combined: the changes in gastric content, gastric emptying, and the ensuing blood responses and appetite. The use of gastric image texture measures provides novel insight into differences in casein coagulation properties that cannot be measured in vivo otherwise. Limitations include the fact that this study was conducted in healthy, young males for minimizing variability in the outcomes, which limits generalizability. Also, the sample size was slightly lower than it should have been (*n* = 18 compared with *n* = 20) based on an updated sample size calculation for the final goat MCC used. This number of subjects is common in both AA and GE studies (e.g., papers on AA availability studies between 10 and 15 subjects [41,[49], [50], [51], [52]]). Additionally, in a review of MRI gastric emptying studies, the 2-treatment crossover studies ranged from 3 to 20 participants, with an average of 15 participants [53]. The lack of difference in gastric emptying was very convincing (goat GE-t50 = 80 ± 25 min compared with cow MC GE-t50 = 85 ± 24 min, *P* = 0.395). Therefore, we believe that a larger sample size would not have resulted in a different conclusion; if a small difference were detected, it would probably be biologically irrelevant.

We here focused on the casein fractions from cow and goat milk, to be able to study casein coagulation in the absence of interaction effects. Future research should explore casein coagulation within a food matrix, as this could significantly influence protein digestion, involving interactions with components like fat [54].

In conclusion, cow MC and goat MC show a difference in coagulation as inferred by AA concentrations and supported by image texture analysis in vivo in humans. This difference in coagulation did not influence overall gastric emptying or the emptied fraction of the liquid and coagulum volume. Therefore, gastric emptying was not the main driver of AA differences. This warrants further research to examine differences in casein coagulation in vivo in the food matrix and how this may affect the digestion of milk products, such as infant formula or medical nutrition. This may help to determine the optimal use of cow and goat milk and their protein fractions.<END ARTICLE>

## Author contributions

The authors’ responsibilities were as follows – WR, GC, PAMS: designed the research; EJMvE: conducted the research, analyzed the data, and drafted the article; WR, LP: helped interpreting results; GC, WR, PAMS: revised the manuscript critically for important intellectual content; PAMS: primary responsibility for final content; and all authors: read and approved the final manuscript.

## Data availability

Data described in the manuscript will be made available on reasonable request.

## Funding

This study was funded by Ausnutria Dairy Corporation Ltd., and WR and LP were employed by Ausnutria Dairy Corporation Ltd. They were not involved in performing the data collection and analysis of the data. They provided comments on a draft version of the article. There were no restrictions regarding publication.

## Conflict of interest

WR and LP were employed as scientists at Ausnutria Dairy Corporation Ltd during the study. The other authors declare that they have no known competing financial interests or personal relationships that could have appeared to influence the work reported in this article.
