# Supplementary material for: Gastric Digestion and Changes in Serum Amino Acid Concentrations after Consumption of Casein from Cow and Goat Milk: A Randomized Crossover Trial in Healthy Males
Source: J Nutr. 2025 Aug 5;155(10):3374–83. doi: 10.1016/j.tjnut.2025.07.025 (PMC12799461; doi:10.1016/j.tjnut.2025.07.025)
Supplement: Multimedia component 1 [file mmc1.docx]

**Supplementary information**

**Supplemental table 1.** *Components of goat and cow milk-derived casein drinks per 300 ml*

| Component | Goat milk-derived protein drink | Cow milk-derived protein drink |
| --- | --- | --- |
| Goat MCC^1^ powder (g) | 49.2 | - |
| Cow MCC powder (g) | - | 38.1 |
| Cow UF^2^ permeate powder (g) | - | 10.1 |
| Water (g) | 248 | 249 |
| Vanilla powder (g) | 3.00 | 3.00 |
| Total (g) | 300.2 | 300.2 |
| *^1^Micellar casein concentrate*  *^2^Ultrafiltration* | | |

**
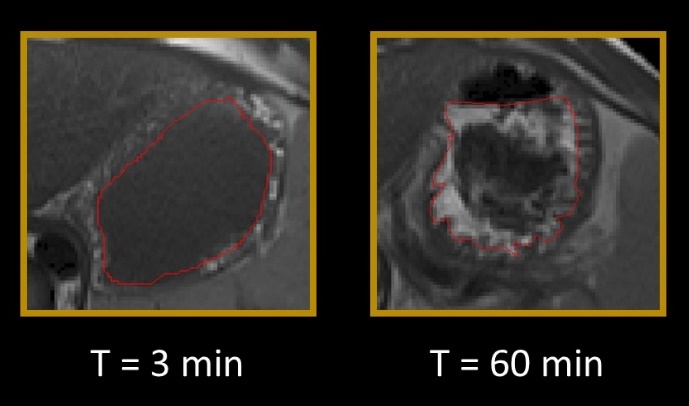
**

**Supplemental figure 1.** An example of stomachs with and without coagulation and their corresponding image texture measures


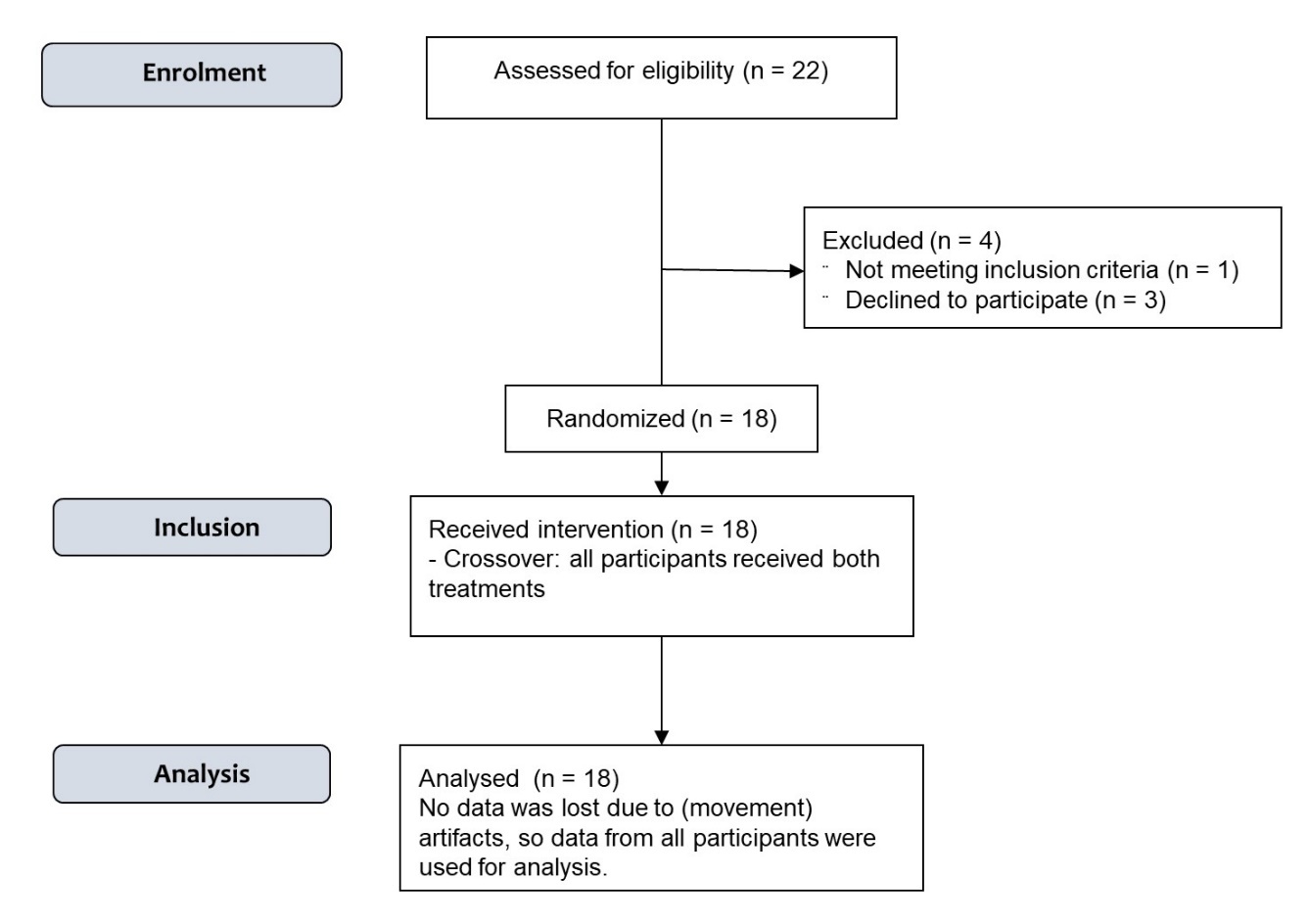


**Supplemental figure 2.** Study flow

**
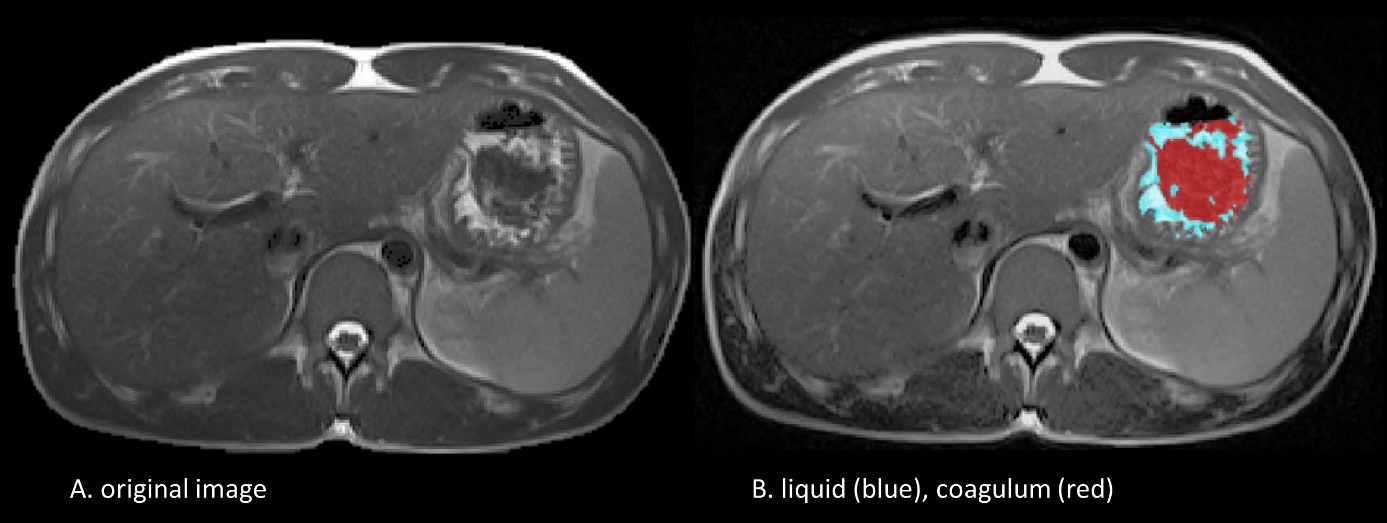
**

**Supplemental figure 3.** Examples of T2-weighted magnetic resonance images showing cross-sections through a stomach at T = 60 min with A showing the original image and B the voxels of stomach content colored: liquid as blue and semi-solid as red after applying the thresholding method.

| 1. **Hunger (from 0 to 100)** | | | | | | |
| --- | --- | --- | --- | --- | --- | --- |
| Type of protein drink | Time of MRI scan continous | Mean | Std. Error | df | 95% Confidence Interval | |
|  |  |  |  |  | Lower Bound | Upper Bound |
| Goat protein | 3 | 39.167 | 3.883 | 68.968 | 31.420 | 46.914 |
|  | 10 | 44.803 | 3.951 | 72.983 | 36.927 | 52.678 |
|  | 20 | 45.833 | 3.883 | 68.968 | 38.086 | 53.580 |
|  | 30 | 48.920 | 3.951 | 72.983 | 41.045 | 56.795 |
|  | 40 | 50.979 | 3.951 | 72.983 | 43.104 | 58.854 |
|  | 50 | 53.056 | 3.883 | 68.968 | 45.308 | 60.803 |
|  | 60 | 58.525 | 3.950 | 72.945 | 50.652 | 66.398 |
| Cow protein | 3 | 38.663 | 3.953 | 73.032 | 30.784 | 46.542 |
|  | 10 | 45.722 | 3.953 | 73.032 | 37.843 | 53.601 |
|  | 20 | 47.781 | 3.953 | 73.032 | 39.902 | 55.660 |
|  | 30 | 50.134 | 3.953 | 73.032 | 42.255 | 58.013 |
|  | 40 | 53.957 | 3.953 | 73.032 | 46.078 | 61.836 |
|  | 50 | 54.840 | 3.953 | 73.032 | 46.961 | 62.719 |
|  | 60 | 55.957 | 4.306 | 95.021 | 47.409 | 64.505 |
|  | | | | | | |

**Supplemental table 2.** *All results per time point*

| 1. **Fullness (from 0 to 100)** | | | | | | |
| --- | --- | --- | --- | --- | --- | --- |
| Type of protein drink | Time of MRI scan continous | Mean | Std. Error | df | 95% Confidence Interval | |
|  |  |  |  |  | Lower Bound | Upper Bound |
| Goat protein | 3 | 51.389 | 4.001 | 40.018 | 43.303 | 59.475 |
|  | 10 | 47.091 | 4.048 | 41.784 | 38.921 | 55.262 |
|  | 20 | 47.500 | 4.001 | 40.018 | 39.414 | 55.586 |
|  | 30 | 43.856 | 4.048 | 41.784 | 35.686 | 52.026 |
|  | 40 | 43.562 | 4.048 | 41.784 | 35.392 | 51.732 |
|  | 50 | 39.167 | 4.001 | 40.018 | 31.080 | 47.253 |
|  | 60 | 35.147 | 4.047 | 41.755 | 26.978 | 43.315 |
| Cow protein | 3 | 53.813 | 4.049 | 41.832 | 45.640 | 61.986 |
|  | 10 | 51.754 | 4.049 | 41.832 | 43.581 | 59.927 |
|  | 20 | 48.519 | 4.049 | 41.832 | 40.346 | 56.692 |
|  | 30 | 44.990 | 4.049 | 41.832 | 36.816 | 53.163 |
|  | 40 | 43.813 | 4.049 | 41.832 | 35.640 | 51.986 |
|  | 50 | 43.813 | 4.049 | 41.832 | 35.640 | 51.986 |
|  | 60 | 41.100 | 4.295 | 51.868 | 32.480 | 49.720 |
|  | | | | | | |
| 1. **Desire to eat (from 0 to 100)** | | | | | | |
| Type of protein drink | Time of MRI scan continous | Mean | Std. Error | df | 95% Confidence Interval | |
|  |  |  |  |  | Lower Bound | Upper Bound |
| Goat protein | 3 | 43.056 | 3.861 | 52.058 | 35.307 | 50.804 |
|  | 10 | 49.896 | 3.918 | 54.826 | 42.042 | 57.749 |
|  | 20 | 50.556 | 3.861 | 52.058 | 42.807 | 58.304 |
|  | 30 | 52.837 | 3.918 | 54.826 | 44.984 | 60.690 |
|  | 40 | 57.837 | 3.918 | 54.826 | 49.984 | 65.690 |
|  | 50 | 56.389 | 3.861 | 52.058 | 48.641 | 64.137 |
|  | 60 | 63.596 | 3.917 | 54.789 | 55.744 | 71.447 |
| Cow protein | 3 | 55.059 | 3.920 | 54.884 | 47.202 | 62.916 |
|  | 10 | 55.647 | 3.920 | 54.884 | 47.790 | 63.504 |
|  | 20 | 59.765 | 3.920 | 54.884 | 51.908 | 67.622 |
|  | 30 | 61.530 | 3.920 | 54.884 | 53.673 | 69.386 |
|  | 40 | 63.000 | 3.920 | 54.884 | 55.143 | 70.857 |
|  | 50 | 62.706 | 3.920 | 54.884 | 54.849 | 70.563 |
|  | 60 | 66.108 | 4.218 | 70.476 | 57.697 | 74.519 |
|  | | | | | | |

| 1. **Prospective consumption (from 0 to 100)** | | | | | | |
| --- | --- | --- | --- | --- | --- | --- |
| Type of protein drink | Time of MRI scan continous | Mean | Std. Error | df | 95% Confidence Interval | |
|  |  |  |  |  | Lower Bound | Upper Bound |
| Goat protein | 3 | 52.500 | 3.652 | 53.690 | 45.178 | 59.822 |
|  | 10 | 53.256 | 3.707 | 56.572 | 45.832 | 60.679 |
|  | 20 | 53.889 | 3.652 | 53.690 | 46.566 | 61.211 |
|  | 30 | 58.844 | 3.707 | 56.572 | 51.420 | 66.268 |
|  | 40 | 57.962 | 3.707 | 56.572 | 50.538 | 65.385 |
|  | 50 | 61.389 | 3.652 | 53.690 | 54.066 | 68.711 |
|  | 60 | 64.648 | 3.706 | 56.534 | 57.226 | 72.069 |
| Cow protein | 3 | 53.813 | 3.708 | 56.631 | 46.386 | 61.240 |
|  | 10 | 57.931 | 3.708 | 56.631 | 50.504 | 65.358 |
|  | 20 | 57.637 | 3.708 | 56.631 | 50.210 | 65.064 |
|  | 30 | 62.637 | 3.708 | 56.631 | 55.210 | 70.064 |
|  | 40 | 62.048 | 3.708 | 56.631 | 54.621 | 69.475 |
|  | 50 | 65.284 | 3.708 | 56.631 | 57.857 | 72.711 |
|  | 60 | 62.137 | 3.994 | 72.828 | 54.176 | 70.098 |
|  | | | | | | |
| 1. **Thirst (from 0 to 100)** | | | | | | |
| Type of protein drink | Time of MRI scan continous | Mean | Std. Error | df | 95% Confidence Interval | |
|  |  |  |  |  | Lower Bound | Upper Bound |
| Goat protein | 3 | 47.500 | 6.165 | 23.488 | 34.762 | 60.238 |
|  | 10 | 50.531 | 6.192 | 23.896 | 37.749 | 63.313 |
|  | 20 | 51.389 | 6.165 | 23.488 | 38.651 | 64.127 |
|  | 30 | 52.590 | 6.192 | 23.896 | 39.808 | 65.371 |
|  | 40 | 53.766 | 6.192 | 23.896 | 40.985 | 66.548 |
|  | 50 | 55.556 | 6.165 | 23.488 | 42.818 | 68.293 |
|  | 60 | 55.965 | 6.191 | 23.888 | 43.184 | 68.746 |
| Cow protein | 3 | 41.809 | 6.193 | 23.910 | 29.025 | 54.592 |
|  | 10 | 47.985 | 6.193 | 23.910 | 35.202 | 60.769 |
|  | 20 | 51.809 | 6.193 | 23.910 | 39.025 | 64.592 |
|  | 30 | 52.985 | 6.193 | 23.910 | 40.202 | 65.769 |
|  | 40 | 53.573 | 6.193 | 23.910 | 40.790 | 66.357 |
|  | 50 | 54.750 | 6.193 | 23.910 | 41.966 | 67.533 |
|  | 60 | 54.902 | 6.337 | 26.180 | 41.879 | 67.924 |
|  | | | | | | |

| **F. Glucose (mmol/l)** | | | | | | | | | | | | |
| --- | --- | --- | --- | --- | --- | --- | --- | --- | --- | --- | --- | --- |
| Type of protein drink | | Time_blood_draws | | Mean | | Std. Error | | df | 95% Confidence Interval | | | |
|  |  |  |  |  |  |  |  |  | Lower Bound | | Upper Bound | |
| Goat protein | | 15 | | 5.741 | | .123 | | 38.292 | 5.492 | | 5.989 | |
|  |  | 30 | | 5.853 | | .123 | | 38.292 | 5.605 | | 6.102 | |
|  |  | 60 | | 5.053 | | .123 | | 38.292 | 4.805 | | 5.302 | |
|  |  | 90 | | 5.047 | | .123 | | 38.292 | 4.799 | | 5.296 | |
|  |  | 120 | | 5.147 | | .123 | | 38.292 | 4.899 | | 5.396 | |
|  |  | 180 | | 5.148 | | .124 | | 40.103 | 4.897 | | 5.399 | |
|  |  | 240 | | 5.167 | | .128 | | 44.598 | 4.909 | | 5.425 | |
| Cow protein | | 15 | | 5.667 | | .123 | | 38.254 | 5.419 | | 5.915 | |
|  |  | 30 | | 5.636 | | .123 | | 38.254 | 5.387 | | 5.884 | |
|  |  | 60 | | 4.883 | | .124 | | 40.081 | 4.632 | | 5.135 | |
|  |  | 90 | | 5.093 | | .123 | | 38.256 | 4.844 | | 5.341 | |
|  |  | 120 | | 5.227 | | .124 | | 40.069 | 4.976 | | 5.479 | |
|  |  | 180 | | 5.106 | | .126 | | 42.180 | 4.851 | | 5.360 | |
|  |  | 240 | | 5.161 | | .124 | | 40.069 | 4.909 | | 5.412 | |
|  | | | | | | | | | | | | |
| 1. **FFA (mmol/l)** | | | | | | | | | | | | |
| Type of protein drink | Time_blood_draws | | Mean | | Std. Error | | df | | | 95% Confidence Interval | | |
|  |  |  |  |  |  |  |  |  |  | Lower Bound | | Upper Bound |
| Goat protein | 15 | | .204 | | .035 | | 174.725 | | | .135 | | .273 |
|  | 30 | | .119 | | .035 | | 174.683 | | | .050 | | .188 |
|  | 60 | | .069 | | .034 | | 167.965 | | | .002 | | .136 |
|  | 90 | | .127 | | .034 | | 167.965 | | | .060 | | .194 |
|  | 120 | | .124 | | .034 | | 167.965 | | | .057 | | .191 |
|  | 180 | | .192 | | .034 | | 167.965 | | | .125 | | .259 |
|  | 240 | | .342 | | .037 | | 188.747 | | | .270 | | .415 |
| Cow protein | 15 | | .285 | | .034 | | 167.965 | | | .218 | | .352 |
|  | 30 | | .150 | | .034 | | 167.965 | | | .083 | | .217 |
|  | 60 | | .076 | | .034 | | 167.965 | | | .009 | | .143 |
|  | 90 | | .103 | | .034 | | 167.965 | | | .036 | | .170 |
|  | 120 | | .126 | | .034 | | 167.965 | | | .059 | | .193 |
|  | 180 | | .178 | | .035 | | 174.683 | | | .109 | | .246 |
|  | 240 | | .251 | | .034 | | 167.965 | | | .184 | | .318 |
|  | | | | | | | | | | | | |

| 1. **Insulin (mIU/L)** | | | | | | | | | | | | | | | | | | | | | | | | | | | |
| --- | --- | --- | --- | --- | --- | --- | --- | --- | --- | --- | --- | --- | --- | --- | --- | --- | --- | --- | --- | --- | --- | --- | --- | --- | --- | --- | --- |
| Type of protein drink | | | Time_blood_draws | | | | | Mean | | | Std. Error | | | | | df | | | | | 95% Confidence Interval | | | | | | |
|  |  |  |  |  |  |  |  |  |  |  |  |  |  |  |  |  |  |  |  |  | Lower Bound | | | | Upper Bound | | |
| Goat protein | | | 15 | | | | | 14.822 | | | 1.745 | | | | | 112.748 | | | | | 11.363 | | | | 18.280 | | |
|  |  |  | 30 | | | | | 18.345 | | | 1.710 | | | | | 106.590 | | | | | 14.956 | | | | 21.734 | | |
|  |  |  | 60 | | | | | 11.880 | | | 1.710 | | | | | 106.590 | | | | | 8.491 | | | | 15.269 | | |
|  |  |  | 90 | | | | | 9.445 | | | 1.710 | | | | | 106.590 | | | | | 6.056 | | | | 12.834 | | |
|  |  |  | 120 | | | | | 9.057 | | | 1.710 | | | | | 106.590 | | | | | 5.668 | | | | 12.446 | | |
|  |  |  | 180 | | | | | 5.842 | | | 1.710 | | | | | 106.590 | | | | | 2.453 | | | | 9.232 | | |
|  |  |  | 240 | | | | | 5.681 | | | 1.829 | | | | | 126.917 | | | | | 2.062 | | | | 9.300 | | |
| Cow protein | | | 15 | | | | | 17.837 | | | 1.710 | | | | | 106.590 | | | | | 14.447 | | | | 21.226 | | |
|  |  |  | 30 | | | | | 20.316 | | | 1.710 | | | | | 106.590 | | | | | 16.927 | | | | 23.705 | | |
|  |  |  | 60 | | | | | 9.091 | | | 1.710 | | | | | 106.590 | | | | | 5.702 | | | | 12.480 | | |
|  |  |  | 90 | | | | | 8.421 | | | 1.710 | | | | | 106.590 | | | | | 5.032 | | | | 11.810 | | |
|  |  |  | 120 | | | | | 8.196 | | | 1.710 | | | | | 106.590 | | | | | 4.806 | | | | 11.585 | | |
|  |  |  | 180 | | | | | 5.820 | | | 1.745 | | | | | 112.748 | | | | | 2.361 | | | | 9.278 | | |
|  |  |  | 240 | | | | | 4.297 | | | 1.745 | | | | | 112.748 | | | | | .839 | | | | 7.755 | | |
|  | | | | | | | | | | | | | | | | | | | | | | | | | | | |
| 1. **Triglyceride (mmol/l)** | | | | | | | | | | | | | | | | | | | | | | | | | | | |
| Type of protein drink | | | | Time_blood_draws | | | | | | Mean | | Std. Error | | | | | df | | | 95% Confidence Interval | | | | | | | |
|  |  |  |  |  |  |  |  |  |  |  |  |  |  |  |  |  |  |  |  | Lower Bound | | | | Upper Bound | | | |
| Goat protein | | | | 15 | | | | | | .874 | | .068 | | | | | 25.650 | | | .734 | | | | 1.013 | | | |
|  |  |  |  | 30 | | | | | | .861 | | .068 | | | | | 25.650 | | | .721 | | | | 1.001 | | | |
|  |  |  |  | 60 | | | | | | .889 | | .068 | | | | | 25.650 | | | .749 | | | | 1.028 | | | |
|  |  |  |  | 90 | | | | | | .938 | | .068 | | | | | 25.650 | | | .799 | | | | 1.078 | | | |
|  |  |  |  | 120 | | | | | | .859 | | .068 | | | | | 25.650 | | | .719 | | | | .998 | | | |
|  |  |  |  | 180 | | | | | | .901 | | .068 | | | | | 26.481 | | | .760 | | | | 1.041 | | | |
|  |  |  |  | 240 | | | | | | .958 | | .069 | | | | | 27.465 | | | .816 | | | | 1.099 | | | |
| Cow protein | | | | 15 | | | | | | .787 | | .067 | | | | | 24.901 | | | .648 | | | | .925 | | | |
|  |  |  |  | 30 | | | | | | .781 | | .067 | | | | | 24.901 | | | .642 | | | | .919 | | | |
|  |  |  |  | 60 | | | | | | .797 | | .067 | | | | | 24.901 | | | .658 | | | | .935 | | | |
|  |  |  |  | 90 | | | | | | .847 | | .067 | | | | | 24.901 | | | .709 | | | | .986 | | | |
|  |  |  |  | 120 | | | | | | .819 | | .067 | | | | | 24.901 | | | .681 | | | | .958 | | | |
|  |  |  |  | 180 | | | | | | .844 | | .068 | | | | | 25.617 | | | .705 | | | | .984 | | | |
|  |  |  |  | 240 | | | | | | .887 | | .068 | | | | | 25.617 | | | .748 | | | | 1.027 | | | |
|  | | | | | | | | | | | | | | | | | | | | | | | | | | | |
| 1. **Gastric volume (ml)** | | | | | | | | | | | | | | | | | | | | | | | | | | | |
| Type of protein drink | | | | | Time of MRI scan continous | | | | Mean | | | | | | Std. Error | | | | df | | | | 95% Confidence Interval | | | | |
|  |  |  |  |  |  |  |  |  |  |  |  |  |  |  |  |  |  |  |  |  |  |  | Lower Bound | | | | Upper Bound |
| Goat protein | | | | | 3 | | | | 293.717 | | | | | | 7.329 | | | | 242 | | | | 279.279 | | | | 308.155 |
|  |  |  |  |  | 10 | | | | 277.395 | | | | | | 7.120 | | | | 242 | | | | 263.371 | | | | 291.419 |
|  |  |  |  |  | 20 | | | | 243.540 | | | | | | 7.120 | | | | 242 | | | | 229.516 | | | | 257.564 |
|  |  |  |  |  | 30 | | | | 222.542 | | | | | | 7.120 | | | | 242 | | | | 208.518 | | | | 236.566 |
|  |  |  |  |  | 40 | | | | 201.813 | | | | | | 7.329 | | | | 242 | | | | 187.376 | | | | 216.251 |
|  |  |  |  |  | 50 | | | | 188.282 | | | | | | 7.329 | | | | 242 | | | | 173.844 | | | | 202.720 |
|  |  |  |  |  | 60 | | | | 172.923 | | | | | | 7.120 | | | | 242 | | | | 158.899 | | | | 186.947 |
| Cow protein | | | | | 3 | | | | 292.409 | | | | | | 7.330 | | | | 242 | | | | 277.970 | | | | 306.847 |
|  |  |  |  |  | 10 | | | | 270.986 | | | | | | 7.324 | | | | 242 | | | | 256.559 | | | | 285.413 |
|  |  |  |  |  | 20 | | | | 236.494 | | | | | | 7.119 | | | | 242 | | | | 222.471 | | | | 250.517 |
|  |  |  |  |  | 30 | | | | 217.812 | | | | | | 7.326 | | | | 242 | | | | 203.382 | | | | 232.243 |
|  |  |  |  |  | 40 | | | | 202.482 | | | | | | 7.557 | | | | 242 | | | | 187.597 | | | | 217.368 |
|  |  |  |  |  | 50 | | | | 196.346 | | | | | | 7.330 | | | | 242 | | | | 181.908 | | | | 210.784 |
|  |  |  |  |  | 60 | | | | 189.046 | | | | | | 7.330 | | | | 242 | | | | 174.607 | | | | 203.484 |
|  | | | | | | | | | | | | | | | | | | | | | | | | | | | |
| 1. **EAA** | | | | | | | | | | | | | | | | | | | | | | | | | | | |
| Type of protein drink | | Time_blood_draws | | | | | Mean | | | | | | | Std. Error | | | | df | | | | 95% Confidence Interval | | | | | |
|  |  |  |  |  |  |  |  |  |  |  |  |  |  |  |  |  |  |  |  |  |  | Lower Bound | | | | Upper Bound | |
| Goat protein | | 15 | | | | | 1830,126 | | | | | | | 95,487 | | | | 32,730 | | | | 1635,794 | | | | 2024,457 | |
|  |  | 30 | | | | | 2067,606 | | | | | | | 94,646 | | | | 31,636 | | | | 1874,732 | | | | 2260,481 | |
|  |  | 60 | | | | | 1968,505 | | | | | | | 94,646 | | | | 31,636 | | | | 1775,631 | | | | 2161,379 | |
|  |  | 90 | | | | | 1951,531 | | | | | | | 94,646 | | | | 31,636 | | | | 1758,657 | | | | 2144,405 | |
|  |  | 120 | | | | | 1969,835 | | | | | | | 95,492 | | | | 32,736 | | | | 1775,496 | | | | 2164,175 | |
|  |  | 180 | | | | | 1866,685 | | | | | | | 95,492 | | | | 32,736 | | | | 1672,346 | | | | 2061,025 | |
|  |  | 240 | | | | | 1924,186 | | | | | | | 96,432 | | | | 33,987 | | | | 1728,210 | | | | 2120,163 | |
| Cow protein | | 15 | | | | | 1961,182 | | | | | | | 93,849 | | | | 30,627 | | | | 1769,682 | | | | 2152,682 | |
|  |  | 30 | | | | | 2280,782 | | | | | | | 93,849 | | | | 30,627 | | | | 2089,282 | | | | 2472,282 | |
|  |  | 60 | | | | | 2061,129 | | | | | | | 93,849 | | | | 30,627 | | | | 1869,629 | | | | 2252,629 | |
|  |  | 90 | | | | | 1989,102 | | | | | | | 93,849 | | | | 30,627 | | | | 1797,602 | | | | 2180,602 | |
|  |  | 120 | | | | | 2057,729 | | | | | | | 93,849 | | | | 30,627 | | | | 1866,229 | | | | 2249,229 | |
|  |  | 180 | | | | | 2025,660 | | | | | | | 94,603 | | | | 31,587 | | | | 1832,860 | | | | 2218,459 | |
|  |  | 240 | | | | | 1974,166 | | | | | | | 94,603 | | | | 31,587 | | | | 1781,367 | | | | 2166,965 | |
|  | | | | | | | | | | | | | | | | | | | | | | | | | | | |
| 1. **NEAA** | | | | | | | | | | | | | | | | | | | | | | | | | | | |
| Type of protein drink | Time_blood_draws | | | | | Mean | | | | | | | Std. Error | | | | | df | | | | 95% Confidence Interval | | | | | |
|  |  |  |  |  |  |  |  |  |  |  |  |  |  |  |  |  |  |  |  |  |  | Lower Bound | | | | Upper Bound | |
| Goat protein | 15 | | | | | 2159,602 | | | | | | | 150,112 | | | | | 25,775 | | | | 1850,912 | | | | 2468,293 | |
|  | 30 | | | | | 2401,483 | | | | | | | 149,273 | | | | | 25,215 | | | | 2094,182 | | | | 2708,784 | |
|  | 60 | | | | | 2377,525 | | | | | | | 149,273 | | | | | 25,215 | | | | 2070,224 | | | | 2684,826 | |
|  | 90 | | | | | 2344,368 | | | | | | | 149,273 | | | | | 25,215 | | | | 2037,067 | | | | 2651,669 | |
|  | 120 | | | | | 2341,645 | | | | | | | 150,117 | | | | | 25,778 | | | | 2032,946 | | | | 2650,344 | |
|  | 180 | | | | | 2109,015 | | | | | | | 150,117 | | | | | 25,778 | | | | 1800,316 | | | | 2417,714 | |
|  | 240 | | | | | 2169,117 | | | | | | | 151,057 | | | | | 26,416 | | | | 1858,853 | | | | 2479,381 | |
| Cow protein | 15 | | | | | 2237,862 | | | | | | | 148,480 | | | | | 24,694 | | | | 1931,871 | | | | 2543,854 | |
|  | 30 | | | | | 2532,147 | | | | | | | 148,480 | | | | | 24,694 | | | | 2226,155 | | | | 2838,138 | |
|  | 60 | | | | | 2450,116 | | | | | | | 148,480 | | | | | 24,694 | | | | 2144,124 | | | | 2756,107 | |
|  | 90 | | | | | 2315,840 | | | | | | | 148,480 | | | | | 24,694 | | | | 2009,848 | | | | 2621,832 | |
|  | 120 | | | | | 2368,578 | | | | | | | 148,480 | | | | | 24,694 | | | | 2062,586 | | | | 2674,569 | |
|  | 180 | | | | | 2286,385 | | | | | | | 149,229 | | | | | 25,187 | | | | 1979,157 | | | | 2593,613 | |
|  | 240 | | | | | 2146,329 | | | | | | | 149,229 | | | | | 25,187 | | | | 1839,101 | | | | 2453,557 | |
|  | | | | | | | | | | | | | | | | | | | | | | | | | | | |

| 1. **BCAA** | | | | | | |
| --- | --- | --- | --- | --- | --- | --- |
| Type of protein drink | Time_blood_draws | Mean | Std. Error | df | 95% Confidence Interval | |
|  |  |  |  |  | Lower Bound | Upper Bound |
| Goat protein | 15 | 1154,534 | 60,399 | 37,690 | 1032,230 | 1276,838 |
|  | 30 | 1317,198 | 59,759 | 36,203 | 1196,025 | 1438,372 |
|  | 60 | 1237,071 | 59,759 | 36,203 | 1115,898 | 1358,245 |
|  | 90 | 1230,304 | 59,759 | 36,203 | 1109,131 | 1351,478 |
|  | 120 | 1241,582 | 60,402 | 37,697 | 1119,271 | 1363,892 |
|  | 180 | 1188,657 | 60,402 | 37,697 | 1066,346 | 1310,967 |
|  | 240 | 1233,374 | 61,116 | 39,399 | 1109,796 | 1356,952 |
| Cow protein | 15 | 1227,973 | 59,153 | 34,838 | 1107,867 | 1348,080 |
|  | 30 | 1451,133 | 59,153 | 34,838 | 1331,027 | 1571,240 |
|  | 60 | 1289,049 | 59,153 | 34,838 | 1168,942 | 1409,156 |
|  | 90 | 1245,822 | 59,153 | 34,838 | 1125,716 | 1365,929 |
|  | 120 | 1292,044 | 59,153 | 34,838 | 1171,938 | 1412,151 |
|  | 180 | 1284,603 | 59,727 | 36,140 | 1163,486 | 1405,720 |
|  | 240 | 1266,632 | 59,727 | 36,140 | 1145,516 | 1387,749 |
|  | | | | | | |

| 1. **Percentage intermediate and solid voxels (coagulum)** | | | | | | |
| --- | --- | --- | --- | --- | --- | --- |
| Treatment | Timepoint | Mean | Std. Error | df | 95% Confidence Interval | |
|  |  |  |  |  | Lower Bound | Upper Bound |
| geit | 3 | 84.197 | 1.559 | 176.459 | 81.119 | 87.274 |
|  | 10 | 79.802 | 1.559 | 176.459 | 76.725 | 82.879 |
|  | 20 | 83.723 | 1.559 | 176.459 | 80.646 | 86.800 |
|  | 30 | 80.530 | 1.559 | 176.459 | 77.453 | 83.607 |
|  | 40 | 78.381 | 1.559 | 176.459 | 75.304 | 81.458 |
|  | 50 | 79.192 | 1.559 | 176.459 | 76.115 | 82.269 |
|  | 60 | 80.151 | 1.559 | 176.459 | 77.074 | 83.229 |
| KOE | 3 | 84.414 | 1.559 | 176.459 | 81.336 | 87.491 |
|  | 10 | 79.758 | 1.559 | 176.459 | 76.681 | 82.836 |
|  | 20 | 81.224 | 1.559 | 176.459 | 78.146 | 84.301 |
|  | 30 | 78.980 | 1.559 | 176.459 | 75.903 | 82.057 |
|  | 40 | 81.069 | 1.559 | 176.459 | 77.992 | 84.146 |
|  | 50 | 80.072 | 1.559 | 176.459 | 76.995 | 83.149 |
|  | 60 | 77.607 | 1.559 | 176.459 | 74.530 | 80.685 |
|  | | | | | | |

| 1. **Homogeneity_weighted** | | | | | | |
| --- | --- | --- | --- | --- | --- | --- |
| Type of protein drink | Time of MRI scan continous | Mean | Std. Error | df | 95% Confidence Interval | |
|  |  |  |  |  | Lower Bound | Upper Bound |
| Goat protein | 3 | .418 | .008 | 250 | .402 | .434 |
|  | 10 | .415 | .008 | 250 | .398 | .431 |
|  | 20 | .398 | .008 | 250 | .382 | .414 |
|  | 30 | .385 | .008 | 250 | .368 | .401 |
|  | 40 | .374 | .008 | 250 | .357 | .390 |
|  | 50 | .365 | .009 | 250.000 | .349 | .382 |
|  | 60 | .358 | .009 | 250 | .341 | .374 |
| Cow protein | 3 | .404 | .008 | 250 | .388 | .420 |
|  | 10 | .407 | .008 | 250 | .390 | .423 |
|  | 20 | .403 | .008 | 250 | .387 | .419 |
|  | 30 | .390 | .008 | 250.000 | .374 | .406 |
|  | 40 | .385 | .008 | 250 | .369 | .402 |
|  | 50 | .379 | .008 | 250 | .362 | .395 |
|  | 60 | .365 | .008 | 250 | .349 | .381 |
|  | | | | | | |

| 1. **Coarseness_weighted** | | | | | | | | | | | | |
| --- | --- | --- | --- | --- | --- | --- | --- | --- | --- | --- | --- | --- |
| Type of protein drink | | Time of MRI scan continous | | Mean | | Std. Error | | df | 95% Confidence Interval | | | |
|  |  |  |  |  |  |  |  |  | Lower Bound | | Upper Bound | |
| Goat protein | | 3 | | .007 | | .001 | | 250 | .006 | | .008 | |
|  |  | 10 | | .007 | | .001 | | 250 | .006 | | .008 | |
|  |  | 20 | | .008 | | .001 | | 250 | .007 | | .009 | |
|  |  | 30 | | .009 | | .001 | | 250 | .008 | | .010 | |
|  |  | 40 | | .010 | | .001 | | 250 | .009 | | .011 | |
|  |  | 50 | | .010 | | .001 | | 250 | .009 | | .011 | |
|  |  | 60 | | .010 | | .001 | | 250 | .009 | | .011 | |
| Cow protein | | 3 | | .007 | | .001 | | 250 | .006 | | .008 | |
|  |  | 10 | | .007 | | .001 | | 250 | .006 | | .008 | |
|  |  | 20 | | .008 | | .001 | | 250 | .007 | | .009 | |
|  |  | 30 | | .009 | | .001 | | 250 | .008 | | .010 | |
|  |  | 40 | | .009 | | .001 | | 250 | .008 | | .010 | |
|  |  | 50 | | .009 | | .001 | | 250 | .008 | | .010 | |
|  |  | 60 | | .010 | | .001 | | 250 | .009 | | .011 | |
|  | | | | | | | | | | | | |
| **Q. Contrast_weighted** | | | | | | | | | | | | |
| Type of protein drink | Time of MRI scan continous | | Mean | | Std. Error | | df | | | 95% Confidence Interval | | |
|  |  |  |  |  |  |  |  |  |  | Lower Bound | | Upper Bound |
| Goat protein | 3 | | .111 | | .009 | | 250.000 | | | .093 | | .128 |
|  | 10 | | .112 | | .009 | | 250 | | | .094 | | .130 |
|  | 20 | | .131 | | .009 | | 250 | | | .113 | | .149 |
|  | 30 | | .147 | | .009 | | 250.000 | | | .129 | | .165 |
|  | 40 | | .179 | | .009 | | 250 | | | .161 | | .196 |
|  | 50 | | .182 | | .009 | | 250 | | | .164 | | .201 |
|  | 60 | | .191 | | .009 | | 250 | | | .173 | | .209 |
| Cow protein | 3 | | .109 | | .009 | | 250 | | | .091 | | .127 |
|  | 10 | | .110 | | .009 | | 250 | | | .092 | | .128 |
|  | 20 | | .129 | | .009 | | 250 | | | .111 | | .147 |
|  | 30 | | .141 | | .009 | | 250.000 | | | .123 | | .158 |
|  | 40 | | .153 | | .009 | | 250 | | | .136 | | .171 |
|  | 50 | | .164 | | .009 | | 250 | | | .146 | | .181 |
|  | 60 | | .175 | | .009 | | 250.000 | | | .157 | | .193 |
|  | | | | | | | | | | | | |
| 1. **Busyness_weighted** | | | | | | | | | | | | |
| Type of protein drink | Time of MRI scan continous | | Mean | | Std. Error | | df | | | 95% Confidence Interval | | |
|  |  |  |  |  |  |  |  |  |  | Lower Bound | | Upper Bound |
| Goat protein | 3 | | .235 | | .014 | | 250.000 | | | .207 | | .263 |
|  | 10 | | .207 | | .014 | | 250 | | | .179 | | .235 |
|  | 20 | | .196 | | .014 | | 250 | | | .168 | | .224 |
|  | 30 | | .169 | | .014 | | 250.000 | | | .141 | | .197 |
|  | 40 | | .159 | | .014 | | 250 | | | .131 | | .187 |
|  | 50 | | .147 | | .015 | | 250 | | | .118 | | .176 |
|  | 60 | | .140 | | .015 | | 250 | | | .111 | | .169 |
| Cow protein | 3 | | .231 | | .014 | | 250 | | | .203 | | .259 |
|  | 10 | | .219 | | .014 | | 250 | | | .191 | | .247 |
|  | 20 | | .205 | | .014 | | 250 | | | .177 | | .233 |
|  | 30 | | .180 | | .014 | | 250.000 | | | .152 | | .208 |
|  | 40 | | .163 | | .014 | | 250 | | | .135 | | .191 |
|  | 50 | | .160 | | .014 | | 250 | | | .132 | | .188 |
|  | 60 | | .149 | | .014 | | 250 | | | .121 | | .177 |

|  |  |
| --- | --- |
|  |  |
|  |  |
| 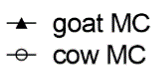 | |

**Supplemental figure 4.** *Mean ± SEM serum concentrations of essential amino acids for cow and goat milk-derived MC drinks. *p < 0.05 placed at the right of the graph denotes a significant treatment effect. Above a data point it denotes a significant time point (post-hoc t-test).*

|  |  |
| --- | --- |
| 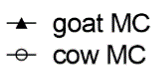 |  |
|  | |

**Supplemental figure 5.** *Mean* ± *SEM serum concentrations of non-essential amino acids* *for cow and goat casein drinks displayed as relative concentrations over time (mean* ± *SEM). *p < 0.05 placed at the right of the graph it denotes a significant treatment effect, above a data point denotes a significant time point (post-hoc test).*

1

**Supplementary figure 6.** *Mean* ± *SEM of image texture measures homogeneity, coarseness, contrast and busyness of stomach content for cow and goat milk-derived casein drinks over time. A higher image contrast reflects a greater degree of structure (possibly coagulation).*

**Supplemental figure 7.** *Mean ± SEM concentrations of glucose (A) and insulin (B) over time after cow and goat milk-derived casein drink ingestion (mmol/L and mIU/L) over time. *p < 0.05 placed at the right of the graph it denotes a significant treatment effect, above a data point denotes a significant time point (post-hoc test).*

**Supplemental figure 8.** *Mean* ± *SEM serum concentrations of free fatty acids (A) and triglycerides (B) for cow and goat milk-derived casein drink ingestion displayed as mmol/L over time. *p < 0.05 placed at the right of the graph it denotes a significant treatment effect, above a data point denotes a significant time point (post-hoc test).*

|  |  | |
| --- | --- | --- |
|  |  | |
| 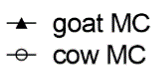 | |  |

***Supplemental figure 9.*** *Mean* ± *SEM appetite ratings, hunger, fullness, desire to eat over time after cow and goat milk-derived MC drink ingestion. *p < 0.05 placed at the right of the graph it denotes a significant treatment effect, above a data point denotes a significant time point (post-hoc test).*


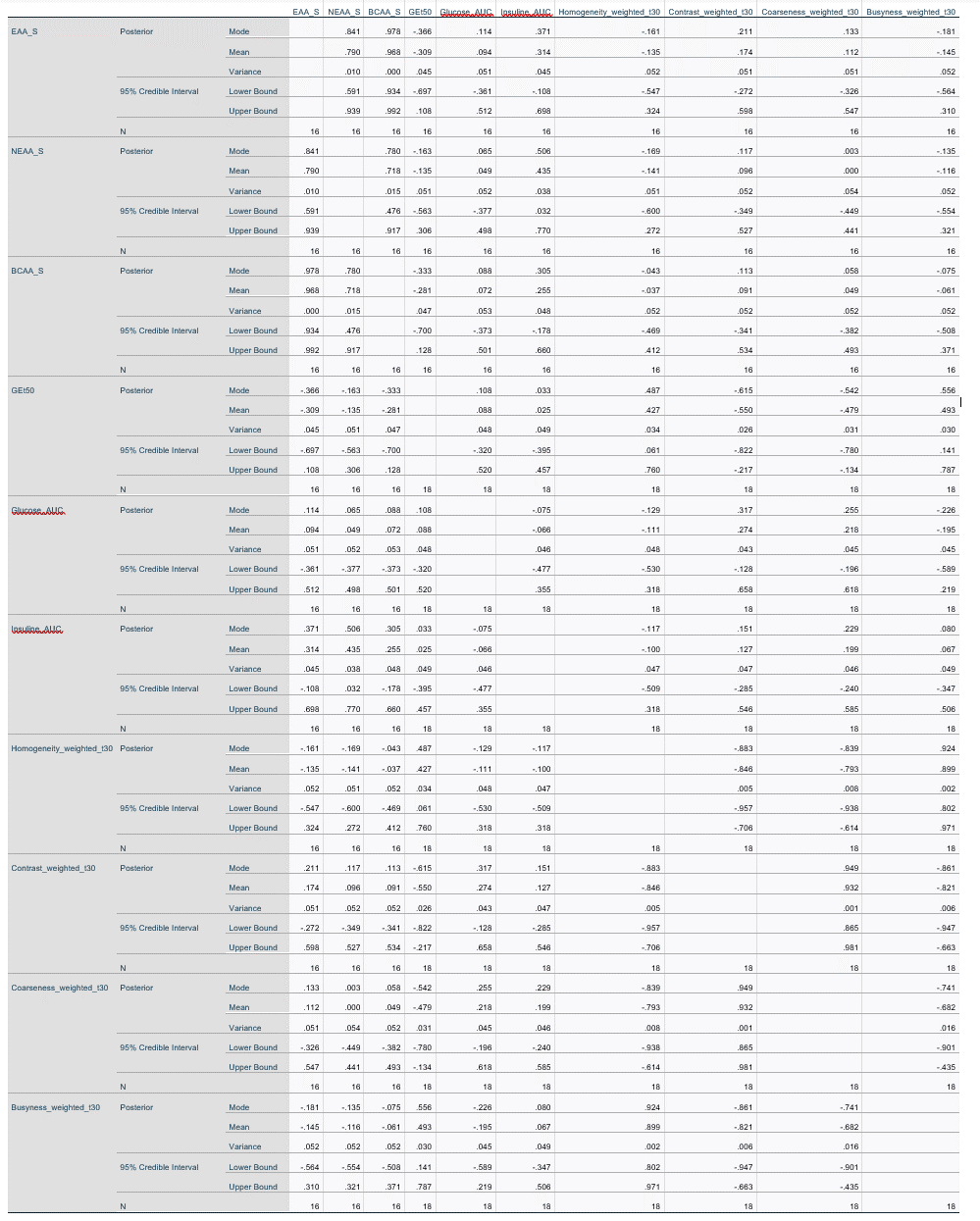


***Supplemental figure 10.*** *Correlations between image texture metrics, blood responses and gastric emptying*
